# Pseudothrombocytopenia due to Platelet Clumping: A Case Report and Brief Review of the Literature

**DOI:** 10.1155/2016/3036476

**Published:** 2016-12-04

**Authors:** Geok Chin Tan, Melissa Stalling, Gretchen Dennis, Maria Nunez, Samir B. Kahwash

**Affiliations:** ^1^Department of Pathology and Laboratory Medicine, Nationwide Children's Hospital, Columbus, OH 43205, USA; ^2^Department of Pathology, National University of Malaysia, 56000 Kuala Lumpur, Malaysia

## Abstract

Platelet clumping is a common laboratory phenomenon that complicates or precludes reporting of platelet count. It is often, but not always, a phenomenon commonly caused by the anticoagulant EDTA. Herein, we discuss a case of a 14-year-old girl who was found to have platelet clumping and discuss the work-up she underwent to investigate her pseudothrombocytopenia.

## 1. Clinical Presentation

A 14-year-old female presented to our hospital with complaint of abdominal pain. Physical examination was unremarkable. She had no bleeding symptoms. The patient's past medical history was significant for Klippel-Feil syndrome and hearing loss. Prior platelet counts had been within normal ranges. The clinical concerns of her current low platelet count included idiopathic thrombocytopenic purpura and bone marrow suppression by a viral illness.

## 2. Laboratory Tests and Findings of Peripheral Blood Smear

**Figure 1:**
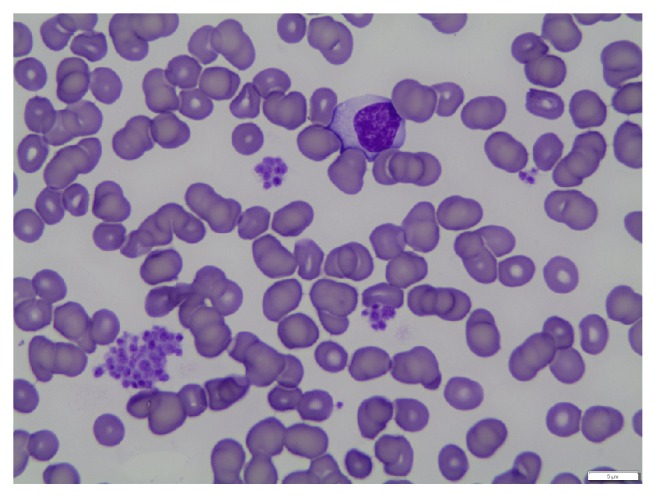
Peripheral blood smear (100x oil).

The platelet count obtained on sample collected in ethylenediaminetetraacetic acid (EDTA) anticoagulant was 80,000 mm^3^. Hemoglobin (Hgb) and WBC were normal. Upon examination of peripheral blood (PB) smear, clumping of platelets was observed. Repeat testing on a sample collected in sodium citrate showed similarly low platelet count and PB smear showed platelet clumps as well. A photomicrograph of her stained PB smear is provided in Figure 1.

EDTA-dependent pseudothrombocytopenia (EDTA-PTCP) was suspected and the clinician was advised to send a sample in a heparin tube. Repeat testing of the new sample presumed to be collected in heparin showed normal platelet count. The CBC data/platelet counts obtained over prior months of past follow-up care were reviewed; see Table 1.

## 3. Discussion

EDTA-dependent pseudothrombocytopenia (EDTA-PTCP) is a common laboratory phenomenon with estimated prevalence of 0.1%–2% in hospitalized patients [1, 2]. It is due to in vitro agglutination of platelets in the blood collection tube caused by IgM/IgG autoantibodies directed against epitopes on platelet surface glycoprotein (GP) IIb/IIIa. EDTA induces a conformational change in GPIIb/IIIa, exposing these epitopes and resulting in platelet agglutination [3]. The use of an alternate anticoagulant, such as citrate or heparin, may be helpful. However, up to 17% of patients with EDTA-PTCP also show this phenomenon with citrate [2, 3].

Bizzaro conducted a large study of EDTA-PTCP cases and found that 83% had antiplatelet antibodies. The phenomenon was not age-related or gender-related, nor was it associated with any particular pathology or use of specific drugs. It showed that EDTA-dependent PTCP is a phenomenon related to the presence of natural autoantibodies with antiplatelet activity and is not associated with any pathological significance [4].

**Figure 2:**
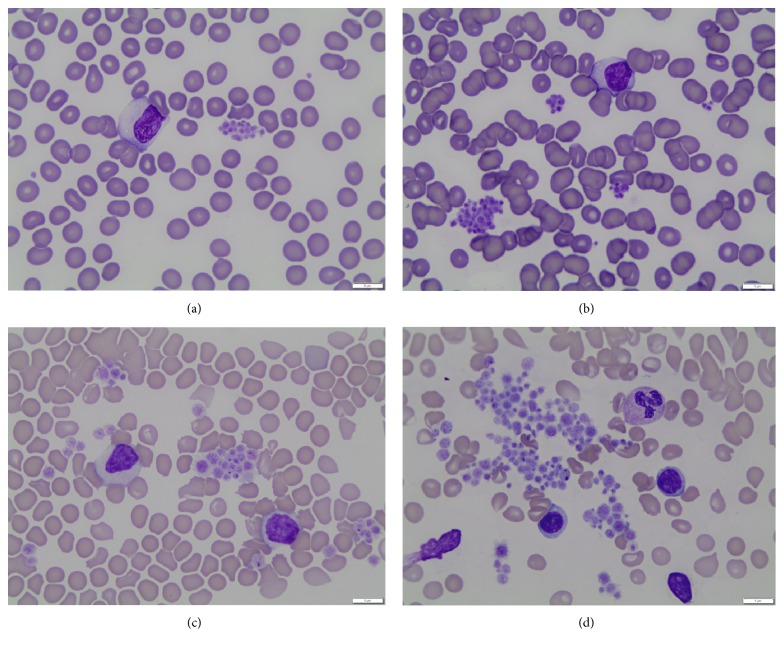
Platelets size and morphology of EDTA-associated clumps in (a) and (b); vWD type 2B-associated clumps in (c) and (d). Note the larger and more variable in size platelets in the latter (all photomicrographs are taken using the same 100x oil lens).

It is important to differentiate EDTA-associated thrombocytopenia from that seen in type 2B von Willebrand disease (vWD type 2B). Kumar and colleagues reported a case of vWD type 2B in a child that was misconstrued as EDTA-PTCP [3]. The patient presented with extensive bruising. CBC showed thrombocytopenia, baseline coagulation profile was normal, and PB smear showed platelet clumping. Due to the severity of bruising, child abuse was suspected as thrombocytopenia was initially misconstrued as being caused by EDTA-related platelet clumping. Further coagulation work-up revealed low von Willebrand factor antigen and ristocetin cofactor activity, and molecular testing confirmed vWD type 2B [3]. The latter is an in vivo consumption of platelet, which results in true thrombocytopenia. Additionally, due to the consumptive nature and compensatory regenerative activity in megakaryocytic cell line, causing a platelet “left shift,” the mean platelet volume (MPV) is increased in vWD type 2B. This morphologic observation may help further separate the two conditions presumptively upon examination of PB smear; refer to Figure 2 for morphologic comparison and Table 2 for comparative features.

Other possible preanalytical factors to consider upon investigating platelet clumps include the collection method, that is, capillary venous or line draws. Capillary collections are prone to clotting and formation of platelet clumps. Viral infection, drugs, and medications, especially chemotherapeutic agents, are all possible inducers of platelet clumping [5, 6].

Clumping can also be due to a combination of more than one of the above factors, and it is possible that a transient viral infection was a confounding cause in our patient (note the atypical lymphocyte suggestive of a viral infection seen in Figure 1 and the CBC results listed in Table 1 showing the fluctuation in WBC, RBC, and Hgb levels coinciding with episodes of clumping and returning to normal levels along with the platelet count).

## 4. Recommendations

From a practical laboratory point of view, investigation of platelet clumping may include the following steps until a nonclumping smear is obtained, noting that Steps 3 and 4 are reserved for the rare instances, where Steps 1 and 2 do not resolve the platelet clumping.

**Step 1 .**

Verify method of blood draw (e.g., finger stick versus venipuncture versus line draw) and exclude collection method related clotting.

**Step 2 .**

Test a blood sample collected in sodium citrate.

If clumping persists, continue to Step 3.

**Step 3 .**

Test a sample collected in heparin. If Step 3 is not possible, proceed to Step 4.

**Step 4 .**

Obtain a sample in ammonium oxalate, and count platelets utilizing a hemocytometer grid, if available, as per described methods [4].

Modern hematology analyzers “flag” platelet clumps, and this should prompt manual verification by examination of a stained PB smear. Reporting platelet counts on samples that show platelet clumps can be a challenge. A recommended approach is not to give a platelet result value for a sample with clumps if the instrument's count is below the lower limits of normal, report the clumping, and recommend one of the steps described above. If the instruments' platelet count of a sample is within or above normal range, a count may be given with an added comment noting the presence of platelet clumps and suggesting that the true count is likely higher than reported.

## Figures and Tables

**Table 1:** Patient's CBC and platelet counts over the period of care and follow-up.

Chronology of follow-up testing	Platelet count, ×10^3^/uL	WBC K/cu mm	RBC M/cu mm	Hgb g/dL	Collection tube additive	Collection method	Collection volume
Follow-up							
Day 1	165	8.8	5.02	15.0	EDTA	Unknown	Unknown
Day 110	106	8.0	5.05	14.6	EDTA	Venipuncture	3 mL
Day 117	87	8.2	4.78	14.1	EDTA	Venipuncture	3 mL
Day 124	88	8.0	5.00	14.7	EDTA	Venipuncture	3 mL
Day 155	Unable to report, platelet clumps	6.6	4.75	14.1	EDTA	Venipuncture	3 mL
Day 156	85	5.0	4.65	13.6	EDTA	Venipuncture	3 mL
Day 188	Unable to report, platelet clumps	4.6	4.70	13.6	EDTA	Venipuncture	3 mL
Day 202^*∗*^	Unable to report, platelet clumps	7.7	4.89	14.4	Na citrate	Venipuncture	2.7 mL
					EDTA		3 mL
Day 216	184	8.4	4.88	14.3	Heparin^*∗∗*^	Venipuncture	3 mL

^*∗*^Encounter described in this case report.

^*∗∗*^Sample collection in a heparin tube was noted in records but could not be verified.

**Table 2:** Comparison between EDTA-associated and vWD type 2B-associated platelet clumping.

Distinguishable features	EDTA-associated	vWD type 2B-associated
Clumping	Due to in vitro process	Due to in vivo process
Bleeding tendency	None	Characteristic
MPV	Normal	Increased due to left shift
Further work-up to confirm platelet clumping	Testing citrate or heparin anticoagulated sample, others	(1) Platelet aggregation studies using low ristocetin concentration (2) Molecular testing (Exon 28 sequencing)
